# Graft-versus-host disease and other cutaneous manifestations in pediatric patients transplanted for Fanconi anemia

**DOI:** 10.1590/1984-0462/2023/41/2022059

**Published:** 2023-07-17

**Authors:** Leane Dhara Dalle Laste, Pâmela Schmidt, Gabriela Araujo Moreira, Janine Horsth Silva, Kerstin Taniguchi Abagge

**Affiliations:** a Universidade Federal do Paraná, Curitiba, PR, Brazil.

**Keywords:** Fanconi anemia, Hematopoietic stem cell transplantation, Haploidentical transplantation, Graft-versus-host disease, Anemia de Fanconi, Transplante de células-tronco hematopoiéticas, Transplante haploidêntico, Doença enxerto-hospedeiro

## Abstract

**Objective:**

The aim of this study was to elaborate a specific protocol for the assessment and early identification of skin lesions in pediatric patients with Fanconi anemia undergoing hematopoietic stem cell transplantation.

**Methods:**

This is a longitudinal, retrospective, and descriptive study. The medical records of 136 pediatric patients with Fanconi anemia who underwent hematopoietic stem cell transplantation between 2008 and 2018 at the Clinical Hospital of the Federal University of Paraná were reviewed. A specific protocol was created for data collection, which included age, sex, skin color, age at diagnosis of Fanconi anemia, transplantation data, family history of consanguinity, and pre- and post-transplant complications. In addition, the data included the presence of graft-versus-host disease of the skin and other organs, its classification, type of lesion, location, and also skin lesions not related to graft-versus-host disease.

**Results:**

Among the skin manifestations in pre-transplant period, café-au-lait spots stood out (32.4%). At least one organ was affected by graft-versus-host disease in 55.1% of patients; the most common involvement being the mouth, followed by the skin. Rash and erythema were the most frequently observed cutaneous manifestations of graft-versus-host disease.

**Conclusion:**

A high prevalence of cutaneous manifestations of the disease was observed, as well as cutaneous manifestations of graft-versus-host disease. The protocol developed gathers relevant and standardized information for the follow-up of patients with Fanconi anemia undergoing hematopoietic stem cell transplantation, ensuring greater reliability of the information, and its implementation will allow the prospective evaluation of patients.

## RESUMO

**Objetivo:**

Elaborar um protocolo específico para a avaliação e identificação precoces de lesões de pele em pacientes pediátricos com anemia falciforme submetidos ao transplante de células-tronco hematopoiéticas.

**Métodos:**

Estudo longitudinal, retrospectivo e descritivo. Foram revisados os prontuários dos pacientes pediátricos com anemia de Fanconi submetidos a transplante de células-tronco hematopoiéticas entre os anos de 2008 e 2018 no Hospital de Clínicas da Universidade Federal do Paraná, totalizando 136 pacientes. Foi criado um protocolo específico para a coleta de dados, que incluiu: idade, sexo, cor, idade ao diagnóstico da anemia de Fanconi, dados do transplante, história familiar de consanguinidade e complicações pré e pós-transplante. Além disso, foram verificados a presença de doença do enxerto contra o hospedeiro da pele e de outros órgãos, sua classificação, tipo de lesão, localização e, também, lesões de pele não relacionadas à doença.

**Resultados:**

Entre as manifestações de pele no período pré-transplante, destacaram-se as manchas café com leite (32,4%). Pelo menos um órgão foi afetado pela doença do enxerto contra o hospedeiro em 55,1% dos pacientes, sendo o acometimento mais comum o de boca, seguido pelo de pele. Exantema e eritema foram as manifestações cutâneas mais frequentemente observadas.

**Conclusões:**

Observou-se alta prevalência de manifestações cutâneas próprias da doença, bem como de manifestações cutâneas de doença do enxerto contra o hospedeiro. O protocolo elaborado reúne informações relevantes e padronizadas para o acompanhamento dos pacientes com anemia de Fanconi submetidos ao transplante, garantindo maior confiabilidade das informações, e sua implementação permitirá a avaliação prospectiva dos pacientes.

## INTRODUCTION

Fanconi anemia (FA) is a rare hereditary disease with an autosomal recessive pattern, with an incidence of approximately 1 in 200,000 births.^1^ It is the most common hereditary cause of bone marrow failure.^2^ The classic triad of the disease is characterized by chromosomal fragility (with increased susceptibility to some cancers, such as those of the skin), malformations, and bone marrow failure.^1^ Typical signs of the disease include congenital and bone deformities, short stature, microcephaly, microphthalmia, gastrointestinal anomalies, auditory anomalies, developmental delay, and hematological findings such as macrocytosis, thrombocytopenia, aplastic anemia, myelodysplastic syndrome, and acute myeloid leukemia.^3^ In addition, changes in skin pigmentation, whether generalized or in patches — especially café-au-lait spots — can present symptoms of FA in up to 40% of patients.^1^ At birth and in early childhood, only physical signs may be present, without hematological involvement, as many patients develop bone marrow failure between 5 and 15 years of age, receiving the diagnosis of FA only at that time.^4^

The most important treatment for these patients is hematopoietic stem cell transplantation (HSCT), which is the only current option capable of prolonging the patients’ lives.^1,5–7^ Graft-versus-host disease (GVHD) is an immune-mediated reaction that causes high morbidity and mortality, and its main determinant in allogeneic HSCT is human leukocyte antigen incompatibility.^8–15^ This disease generates direct cytotoxicity to some organs, especially the skin, gastrointestinal tract, eyes, and liver.^12–16^ GVHD can be classified as acute and chronic. The manifestations of skin disorders are very common and are often the first telltale sign of acute GVHD. They are classically described as erythematous maculopapular eruptions (rash) located on the face, palms of the hands, and soles of the feet. Such skin lesions often spread to the trunk and may progress to erythroderma, affecting a large area of the body.^15^ In chronic GVHD, early manifestations of xerosis, ichthyosis, and keratosis pilaris-like lesions are common, but the most characteristic are poikiloderma, lichenoid lesions, and sclerotic skin changes. Other cutaneous findings commonly observed in chronic GVHD include nail and mucosa alterations and vitiligo-like depigmentation.^17^

The main criteria used for the differential diagnosis between acute and chronic GVHD are those present in the consensus of the National Institute of Health (NIH).^18^ They categorize manifestations according to the likelihood of chronicity of the condition. However, the detail and complexity of this and other protocols do not fully fit all daily medical care needs.^19^ This is because there is a lack of an approach more specific to individual organ involvement. In addition, evaluation of pediatric patients is flawed, as it is usually performed according to the guidelines followed for adults. The main objective of this work was to build a specific protocol for the assessment and early identification of pre- and post-transplant skin lesions in pediatric patients with FA undergoing HSCT so that it would be applicable in outpatient clinics and specialized services. The protocol prioritizes the identification of post-transplant manifestations characteristic of GVHD and allows for early diagnosis and treatment of this condition.

## METHOD

This is a longitudinal, retrospective, and descriptive study approved by the Ethics Committee for Research on Human Beings of Clinical Hospital of the Federal University of Paraná (HC-UFPR), on March 17, 2019, CAAE 07612818.0.0000.0096 and opinion number 3,203.530.

The source population of the study corresponds to pediatric patients (under 18 years of age) with FA undergoing HSCT at the Transplantation Service of HC-UFPR. The registry number of transplanted patients was obtained from an existing database at the Hematopoietic Stem Cell Transplantation Service, and the medical records were requested from the hospital's Medical Archive Service. The medical records of patients who underwent HSCT at HC-UFPR from 2008 to 2018 were evaluated. Based on data obtained retrospectively from the medical records of transplanted patients, a specific protocol was designed in 2018 for the dermatological follow-up of pediatric patients with GVHD of the skin.

For the elaboration of the protocol, scientific articles on the main skin manifestations of GVHD were reviewed, and the NIH classification was used as a basis.^18,20^ Several protocols were tested — by filling in data from medical records — before reaching the final version. The selection of manifestations to be included was made through the observation of the most prevalent manifestations, both in HC-UFPR patients and in the literature. The protocol was constructed in the most practical way possible, in order to facilitate its completion. The complete protocol is available with the corresponding author.

Data were collected such as age, sex, color, age at diagnosis of FA, HSCT data (patient's age at completion, type, number, donor, and percentage of donor cells present after transplantation, through the Variable Number of Tandem Repeats), family history of consanguinity, and pre- and post-transplant complications. In addition, the presence of GVHD of the skin and other organs, grading of the skin GVHD, type of lesion, location, association with GVHD in other sites, and skin lesions not related to GVHD (pre- and post-transplantation) were evaluated.

The obtained data were arranged graphically in a Microsoft Excel^®^ data sheet and analyzed using the statistical analysis software JMP^®^ and Statistica version 10.0 (StatSoft^®^).

Measures of central tendency and dispersion were expressed as means and standard deviation (mean±SD) for continuous variables with symmetrical distribution. Categorical variables were expressed as absolute and relative frequencies. To estimate the difference between continuous variables of symmetric distribution, a Student's t-test and an analysis of variance were applied. Pearson's chi-square test was applied to estimate the difference between categorical variables. Finally, the minimum level of significance of 5% was considered for all tests.

## RESULTS

The study sample consisted of 136 patients, 57 (41.9%) females and 79 (58.1%) males, with a median age of 15.3 (P25-75: 2.28) years at the time of transplantation, with 89 (65.4%) of them white, 46 (33.8%) brown, and 1 (0.7%) black. The distribution of origin of patients in relation to the regions of Brazil was 5.9% in the North, 30.1% in the Northeast, 14% in the Midwest, 21.3% in the Southeast, and 28.7% in the South.

The age at diagnosis was a median of 7 years, ranging from 1 to 14 years. Almost 70% of the transplants were performed from 2012 onward.

In total, 11 (8.1%) patients had more than one transplant, of whom 10 had two and 1 had three. Consanguinity was recorded in 22 (16.2%) cases, and other cases of FA in the family were observed in 12 (8.8%).

In all, 35 (25.5%) patients underwent haploidentical transplantation from 2008 to 2018, with a progressive percentage increase every 4 years: 7.8% of the transplants from 2008 to 2011 and 35.3% of those from 2012 to 2015. From 2016 to 2018, 38.2% of transplants were haploidentical.

**Figure 1:**
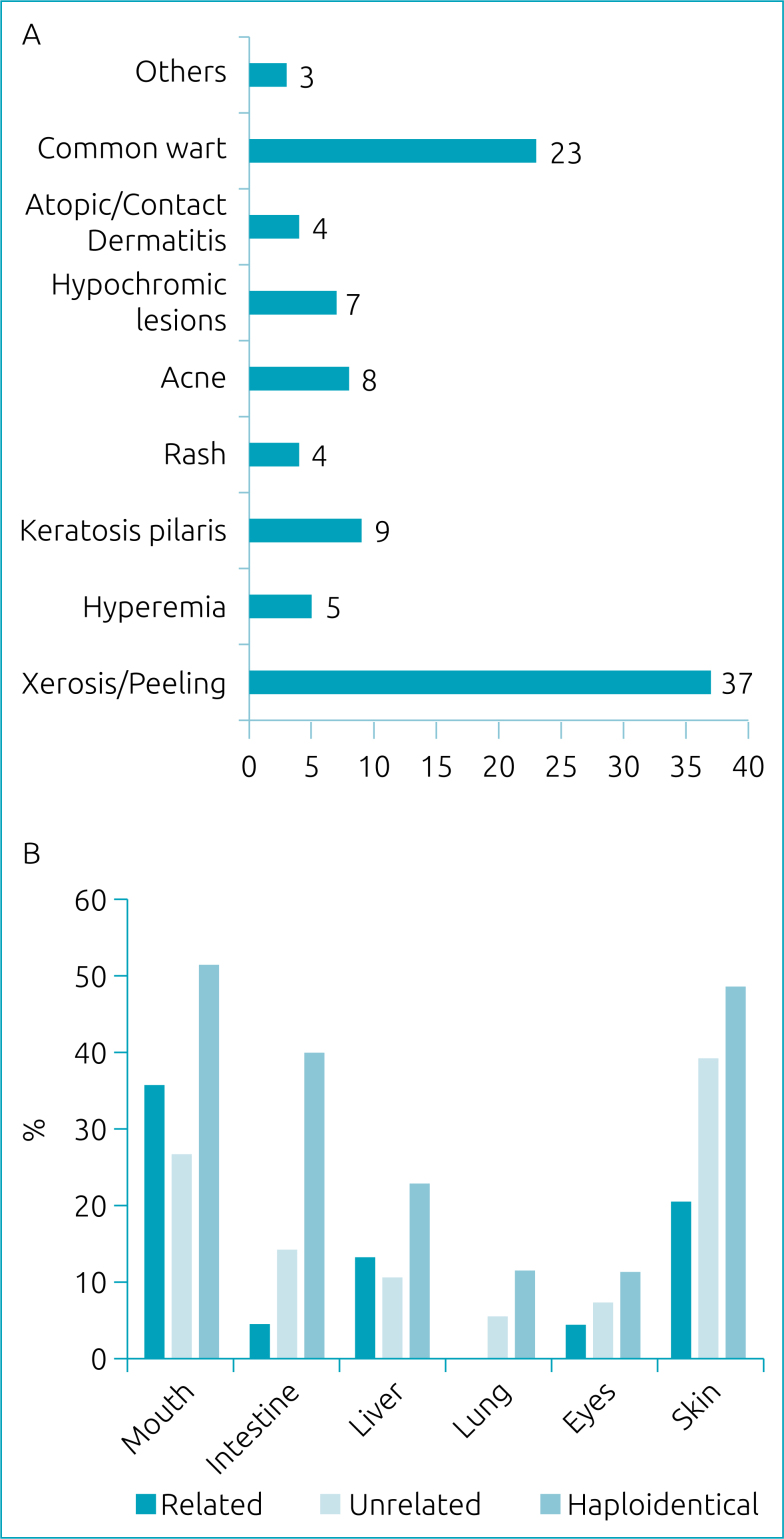
**A.** Frequency of unrelated skin injuries to graft-versus-host disease after transplantation. **B.** Frequency of graft-versus-host disease according to the type of transplantation.

Regarding pre-transplant skin manifestations, café-au-lait spots were present in 32.3% of patients, and hypochromic spots were present in 5.1%. Figure 1A illustrates the frequency distribution of non-GVHD skin lesions observed after HSCT. Xerosis with desquamation was the most prevalent manifestation.

GVHD was present in 55.1% of all transplant patients, with the most frequent involvement being the mouth (36.0%), followed by the skin (35.5%), intestine (17.6%), liver (14.7%), eyes (7.3%), and lungs (5.1%), as can be seen in Table 1.

**Table 1:** Frequency and characteristics of systemic involvement from graft-versus-host disease.

Characteristics		Mouth (%)	Intestine (%)	Liver (%)	Lung (%)	Eyes (%)	Skin (%)
Frequency		49 (36.0)	24 (17.6)	20 (14.7)	7 (5.1)	10 (7.3)	48 (35.5)
Acute		0	9	8	0	0	17 (77.3)
Chronic		32 (23.5)	3	4	4	4	5 (22.7)
National Institute of Health classification							
	1	8	0	0	1	1	1
	2	1	3	4	0	2	7
	3	0	2	1	0	0	10
	4	0	9	3	0	0	3

GVHD: Graft-versus-host disease; National Institute of Health classification: 1: Enough to make the diagnosis; 2: Seen in cGVHD but not diagnostic; 3: It may be part of the presentation of cGVHD if the diagnosis is confirmed by other findings; 4: Seen in both DECHa and cGVHD.

A higher frequency of liver GVHD was observed in haploidentical transplants (p<0.001) and skin GVHD in unrelated and haploidentical transplants (p=0.02). Mouth and lung GVHD were more frequent in haploidentical transplantation, with a borderline significance level (p=0.05 and p=0.07, respectively). The distribution of GVHD frequency according to the type of transplant is shown in Figure 1B.

**Figure 2:**
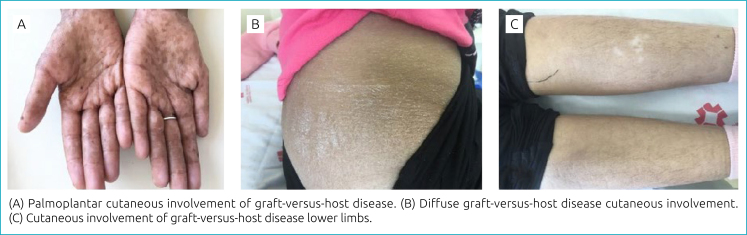
Cutaneous manifestations of graft-versus-host disease.

Rash (23.7%) and erythema (14.8%) were the most frequently observed manifestations of cutaneous GVHD, which can be seen in Figure 2.

In Table 2, it is observed that the rash was present mostly diffusely (77.8%) and in the palmoplantar region (11.1%), and erythema occurred more in the palmoplantar region (38.5%) and limbs (30.8%).

**Table 2:** Cutaneous manifestations of graft-versus-host disease and its body distribution.

Characteristics	Rash (%)	Xerosis (%)	Erythema (%)	Keratosis (%)	Hyperpigmentation (%)	Hypochromia (%)	Eczema	Lichen planus
Frequency	32 (23.7	16 (11.8)	20 (14.8)	16 (11.8)	10 (7.4)	6 (4.4)	5 (3.7)	3 (2.2)
Palmoplantar	2 (11.1)	0	5 (38.5)	0	0	0	0	0
Head	1 (5.5)	3	2 (15.4)	0	0	0	0	0
Trunk	1 (5.5)	1	0	2	2	2	1	0
Limbs	0	0	4 (30.8)	1	3	0	3	0
Diffuse	14 (77.8)	4	2 (15.4)	1	2	2	0	0

## DISCUSSION

In the present study, more than a quarter of the patients underwent haploidentical transplantation. The prevalence of this modality has increased over the years, being higher from 2016 to 2018, compared to other periods from 2008 onward. Previous work carried out at the Bone Marrow Transplantation Service of HC-UFPR showed a survival rate of 73% in 1 year with this type of HSCT, which reinforces its effectiveness for the management of patients with FA, especially after the introduction of new induction protocols before and after transplantation.^21^ However, the incidence of rejection and GVHD represents an obstacle to the success of this type of HSCT,^14^ which was confirmed by the current study, since all types of GVHD analyzed (mouth, intestine, liver, lung, eyes, and skin) were more common in patients who underwent haploidentical transplantation. The early identification of GVHD lesions by the developed protocol could institute early treatment and avoid the therapeutic failure of HSCT.

GVHD is an adverse immunological phenomenon observed after allogeneic HSCT and can be considered an exaggerated and undesirable response to the graft. Its incidence is high, ranging from 40 to 60%,^22^ which is similar to that obtained in the present study (55.1%).

In a previous study carried out at the HC-UFPR, the prevalence of oral GVHD observed among 103 patients transplanted for FA before 2013 was 42%.^23^ This percentage is similar to that found in the present study (36%), in which GVHD of the mouth was followed by involvement of the skin, intestine, liver, eyes, and lung. The considerable incidence of oral GVHD implies a periodic and thorough evaluation of the oral cavity, as an important clinical measure for the diagnosis and prognosis of pediatric transplant patients. Mouth GVHD can be accompanied by dysphagia, xerostomia, and a burning sensation, even causing difficulty in eating and leading to nutritional deficits.

It was observed that 48 (35.5%) children had skin GVHD, but due to the lack of information in the medical records, it was possible to classify only 22 cases: 17 in the acute form and 5 in the chronic form. Most of them were classified as grade III (n=10), affecting more than 50% of the total body surface.

Among the manifestations of acute GVHD of the skin, rash, follicular erythema, epidermolysis, and pruritus stand out. Palmoplantar location is common in children.^17^ These data are corroborated by the results of this study, which showed that the main manifestations were rash and erythema. The rash was present, mostly in a diffuse form and in the palmoplantar region. Erythema, on the other hand, occurred more in the palmoplantar region and limbs. The fact that the diffuse form of the exanthema is the most observed is due to the centrifugal distribution of GVHD skin lesions, which start on the face, neck, and trunk, being more concentrated in these regions and distributed to the limbs. Both exanthema and erythema were predominant in the palmoplantar region, as GVHD can start with isolated cutaneous manifestations of the extremities, preceding other cutaneous manifestations.

It was found that patients with FA from all regions of Brazil have already been treated at the HC-UFPR. It is noteworthy that the South region, where the HC-UFPR is located, is not the first in terms of the total percentage of patients who underwent HSCT for FA between 2008 and 2018. Almost one-third of these patients came from the Northeast region, which confirms the national importance of HC-UFPR as a reference center in FA.

The literature cites a ratio of 1:0.91 (male:female),^19,24–26^ which is confirmed in the present study, with a slight predominance of males.

Regarding the color of patients with FA, information in the literature is scarce. In our survey, it was observed that most of the affected patients were white. However, 50% of the sample was patients from the South and Southeast, regions with a higher prevalence of whites when compared to the rest of the country.

The most common pre-HSCT skin manifestations found in this study were café-au-lait spots, which were present in almost one-third of patients. These data are corroborated by previous studies, which cite these spots as prevalent cutaneous signs of FA.^27–30^ However, it is possible that more patients presented with these skin changes since information from pre-transplant consultations was often not well documented in the medical records. The major limitation of the problems found in our study was the description of the solutions of problems observed and, in some cases, the description of the cases described in our study, which were not observed. The implementation of the protocol was developed as the correct destination of the identified solutions, as well as avoiding the protocol by the identified health professionals.

There were many difficulties during the process of data collection and analysis, due to lack of standardization of records, lack of information in the medical records, and incomplete descriptions of skin lesions. This reinforces the importance of establishing a standard protocol that contains all relevant information for the registration and follow-up of transplant patients.

This study limitations included retrospective data collection and variable or incomplete descriptions of cutaneous manifestations presented by patients with FA.

The developed protocol gathers relevant and standardized information on pre- and post-transplant cutaneous manifestations, including GVHD cutaneous manifestations, for the follow-up of patients with FA undergoing transplantation, ensuring greater reliability of the information, and its implementation will allow the prospective evaluation of patients, which can improve early diagnosis of GVHD and its clinical outcome.

Thus, the present article is innovative in bringing a specific protocol to identify post-transplant skin lesions, especially GVHD, and allow them to compare pre-transplant lesions in order to diagnose GVHD early. The protocol is original, easy to apply, and standardizes the information obtained during pediatric consultations in dermatology and hematology clinics, as well as in bone marrow transplant services, ensuring that the evolution of lesions is monitored and the emergence of new lesions is more easily identified.

This protocol represents an important contribution to early diagnosis, analysis of evolution, duration, response to treatment, and determination of the impact of such manifestations on the quality of life of transplanted patients.

Suggestions for future studies would be to evaluate the impact of the implementation and the effectiveness of the protocol for the diagnosis and early treatment of post-HSCT cutaneous manifestations, especially for GVHD manifestations.
